# Perceptions of individuals regarding barriers to participation in a pulmonary rehabilitation program after hospitalization due to COVID-19: A qualitative study

**DOI:** 10.1371/journal.pone.0294963

**Published:** 2023-11-28

**Authors:** Rafaella Rabelo Polato, Cristino Carneiro Oliveira, Yuri Augusto de Sousa Miranda, Leandro Ferracini Cabral, Carla Malaguti, Anderson José

**Affiliations:** 1 Postgraduate Program in Rehabilitation Sciences and Physical Functional Performance, Federal University of Juiz de Fora, Juiz de Fora, Brazil; 2 Cardiac and Respiratory Physiotherapy Department, Federal University of Juiz de Fora, Juiz de Fora, Brazil; DIT University, INDIA

## Abstract

**Introduction:**

Several individuals with post-COVID-19 syndrome referred for pulmonary rehabilitation did not participate. This study aimed to explore individuals’ barriers to participating in posthospitalization COVID-19 rehabilitation.

**Materials and methods:**

This was a qualitative, multicenter study performed using semistructured interviews. This study included 20 individuals hospitalized for COVID-19 who refused to participate in a pulmonary rehabilitation program at a university hospital.

**Results:**

Individuals reported difficulties accessing the rehabilitation center, mainly due to distance, transport costs and conditions, and lack of companions. Health problems (e.g., surgeries, pain, and mobility difficulties) and lack of time due to work, commuting, and household work were also reported. Another reported theme was not perceiving the need for rehabilitation due to feeling well. Minor themes included the need for more information about rehabilitation and a lack of interest, motivation, and medical encouragement.

**Conclusion:**

Individuals hospitalized for COVID-19 faced several barriers to participating in a pulmonary rehabilitation program. These barriers included difficulties in accessing the rehabilitation center, health problems, lack of time, and the perception that rehabilitation was unnecessary. There is a need for actions to overcome these barriers to make the program available to a larger number of individuals.

## Introduction

Coronavirus disease 2019 (COVID-19) may cause pulmonary and systemic repercussions, leading to persistent symptoms and long-term complications. When these complications persist for more than four weeks from the onset of symptoms, the condition is named post-COVID-19 syndrome [1]. Post-COVID-19 syndrome may reduce functional capacity and independence, causing a substantial social impact and impairing quality of life [2, 3].

Pulmonary rehabilitation is the most effective nonpharmacological therapy to improve functional capacity, quality of life, and exercise capacity and reduce symptoms in individuals with chronic respiratory diseases [4]. These benefits have also been demonstrated in individuals with post-COVID-19 syndrome [5–7]. However, offering pulmonary rehabilitation to individuals with chronic respiratory diseases is challenging due to low rates of referral, participation, and adherence to the treatment [8, 9].

To date, there have been no studies specifically designed to investigate the participation of individuals in a rehabilitation program following COVID-19. The available data, obtained through the enrollment of subjects in randomized clinical trials, reveal that several individuals with post-COVID-19 syndrome, who were referred to rehabilitation, did not participate in the program for reasons that remain unknown [10–12].

Hence, these reasons need to be investigated to adopt facilitating actions to increase participation and provide the benefits of rehabilitation for this population. This study aimed to explore individuals’ barriers to participating in posthospitalization COVID-19 rehabilitation.

## Methods

This qualitative multicenter study used semistructured interviews based on grounded theory [13–15]. Participants were recruited at three public tertiary hospitals in a single city and referred to a pulmonary rehabilitation program at a university hospital between January 2022 and August 2022. This study was approved by the research ethics committee of University Hospital of Federal University of Juiz de Fora (no. 5,215,309), and all participants provided written informed consent. The authors did not have access to information that could identify individual participants after data collection.

The inclusion criteria were individuals aged 18 years or older who were hospitalized for COVID-19 and refused to participate in a pulmonary rehabilitation program after hospital discharge. The rehabilitation program followed a standard model, with sessions twice a week for eight weeks [4]. Each session consisted of 30 minutes of aerobic training (e.g., treadmill walking or cycling) and 20 minutes of muscle resistance training using items such as elastic bands, ankle weights, and dumbbells [4].

The exclusion criteria included cognitive disorders that impeded understanding study procedures and failure to respond to contact attempts. A researcher not involved in the pulmonary rehabilitation program invited individuals to participate in this study via telephone. Six contact attempts were made before excluding the individual. The principle of data saturation determined the sample size [15, 18].

Questions were developed and focused on the main theme of this study to explore perceptions regarding barriers associated with nonparticipation in a pulmonary rehabilitation program. The questions were adapted from a study investigating barriers to participation in a pulmonary rehabilitation program for individuals with chronic obstructive pulmonary disease [16].

### Interview questions

1. Why do you think you were referred to a pulmonary rehabilitation program?
2. I understand that you chose not to participate in the pulmonary rehabilitation program. Can you tell me about this?
3. What was the most important factor that prevented you from participating in the pulmonary rehabilitation program?
4. What information did you receive about the pulmonary rehabilitation program?
5. What do you think a pulmonary rehabilitation program is?
6. Do you think that rehabilitation would provide any benefit for your health?
7. Would you consider participating in a pulmonary rehabilitation program in the future?
8. In the future, for you to participate in a rehabilitation program, what circumstances should change?

The interviews were conducted solely by the first author of this study, a physiotherapist and a master’s student in “Rehabilitation Sciences and Physical Functional Performance”, with experience in caring for patients with chronic respiratory and renal diseases. Semistructured interviews were held face-to-face (at home) or remotely (via telephone). Face-to-face interviews were conducted in the participant’s home, with time available for the interview, seated comfortably in a quiet, closed, and confidential environment, with only the researcher and the participant present. For telephone interviews, it was confirmed that the participant was at home, with time available for the interview, seated comfortably in a quiet, closed and confidential environment without the presence of other people. The researcher was encouraged to take notes during the interviews for subsequent use during the analysis [13].

A single researcher fully transcribed each recorded interview. Demographic data, smoking habits, use of mechanical ventilation during hospitalization, housing situation, education level, family income, distance from home to the rehabilitation center, and Charlson Comorbidity Index score were also collected [17].

### Analysis

A thematic analysis of interviews was performed using line-by-line coding [18], and three rounds of coding were performed. This study utilized data triangulation to corroborate findings and gain diverse perspectives. Two researchers independently examined the interview transcripts, and a third researcher joined the process until a consensus was established [19].

The open coding process commenced concurrently with data collection to create a hierarchical coding framework. As the data collection progressed, the axial coding phase was initiated to further enhance and clarify the connections between overarching themes and their corresponding subthemes. This iterative coding approach allowed for a comprehensive and structured analysis of the data, ensuring that the relationships between different levels of coding were well defined and coherent [13].

A theme describes the various facets of a pattern across the dataset. A subtheme exists ’underneath’ the theme’s umbrella; it shares the same central organizing concept as the theme but concentrates on a specific element [13, 20].

Finally, selective coding explored connections among themes and selected the main category. Theoretical memos were used during analysis to reflect how findings were derived from the data [13–15], and citations were extracted from the transcripts to provide supporting data for each theme. Recruitment and data collection continued until saturation [15, 18, 21]. Quantitative data analysis used the Shapiro‒Wilk test to verify data normality. Continuous variables with a normal distribution are expressed as the means and standard deviations, and those with a nonnormal distribution are described as the medians and 25–75% interquartile ranges. Categorical variables are described as absolute and relative frequencies.

## Results

A total of 104 individuals were referred to the pulmonary rehabilitation program between December 2021 and August 2022. Ninety-nine individuals declined to participate in the rehabilitation program and were considered eligible for inclusion in this study. Among these, 79 individuals were excluded (60 due to contact failure and 19 due to refusal to participate in this study). This left a final sample of 20 individuals. Twelve interviews were conducted face-to-face and eight by telephone, without differences in the identified themes. The interviews lasted ten to twenty minutes. Additionally, the sample was saturated with seventeen interviews, and three additional interviews were performed for confirmation [15]. With this sample, the objectives of this study were achieved, and new information was scarce [15, 18, 21].

The median age was 50 years [25–75%: 36.5–78.5], and there were 15 females and 5 males. One individual lived alone, whereas others lived with families. The characteristics of the individuals are described in Table 1.

**Table 1:** Sample characteristics.

Characteristics	Total (n = 20)
Age, years	55.0 [36.5–78.5]
Female, n (%)	15 (75)
BMI, kg/m^2^	29.4 ± 7.0
Hospitalization, days	6.0 [4.0–11.3]
MV use, n (%)	2 (10)
NIV use, n (%)	9 (45)
Current smoker, n (%)	1 (5)
Charlson Comorbidity Index, points	2.5 [0.0–5.6]
Comorbidities, n (%)	
Hypertension	2 (25)
Diabetes	8 (40)
Others	14 (70)
Housing distance from the rehabilitation center, km	9.0 [7.0–12.6]
Education level, n (%)	
Complete elementary school	11 (55)
Complete high school	7 (35)
Complete higher education	2 (10)
Family income, n (%)	
< US$ 250.00	6 (30)
From US$ 250.00 to US$ 500.00	10 (50)
From US$ 500.00 to US$ 750.00	4 (20)

BMI: body mass index; MV: mechanical ventilation; NIV: noninvasive mechanical ventilation. Values are expressed as the means ± standard deviations, medians [25–75%], or absolute frequencies (%).

### Themes associated with nonparticipation

The themes related to refusal to participate in the pulmonary rehabilitation program were difficulties accessing the rehabilitation center, health problems, lack of time, and not perceiving the need for rehabilitation due to feeling well (Table 2). The following citations were extracted from the transcripts to provide supporting data for each theme.

**Table 2:** Themes, subthemes, and minor themes related to refusal to participate in the pulmonary rehabilitation program.

Themes	Total (n = 20)
**Difficulties accessing the rehabilitation center**	**13 (65)**
Distance	09 (45)
Cost of transport	06 (30)
Lack of companion	03 (15)
Transport conditions	03 (15)
**Health problems**	**10 (50)**
Pain	06 (30)
Preoperative problems	03 (15)
Mobility difficulty	02 (10)
Other	09 (45)
**Lack of time**	**07 (35)**
Work	03 (15)
Commuting time	03 (15)
Household work	02 (10)
**No need perceived**	**05 (25)**
Feeling well	05 (25)
**Minor themes**	**05 (25)**
Demotivation	02 (10)
Need for more information	01 (5)
Lack of interest	01 (5)
Lack of medical encouragement	01 (5)

Values expressed as absolute frequencies (%).

#### Difficulties accessing the rehabilitation center

The primary theme reported by participants was reaching the rehabilitation center, as mentioned by thirteen individuals. Many individuals resided at a considerable distance from the university hospital where the rehabilitation would take place, necessitating the use of multiple public transportation modes, often of poor quality.

Nine individuals indicated distance as a barrier.

“It is too far; I need a taxi. It would be difficult because of the commute.” (Participant 13)

“For me, the problem is that it would be at the university hospital. It would be too far away.” (Participant 1)

“I want to do it, but it needs to be in a closer place.” (Participant 3)

Six individuals had financial difficulties commuting to the rehabilitation center.

“There is no car to take me, and to pay for a vehicle, an Uber or taxi, is very expensive.” (Participant 17)

“I would have to take four buses, two to go and two to return. In addition, the ticket, four tickets would be a bit expensive.” (Participant 19)

Three individuals reported the lack of a companion as a barrier.

“Yes, I can participate if someone takes me.” (Participant 18)

Three individuals reported that transport conditions affected their decision to participate.

“The bus from here to there rumbles and jumps and only God knows what else. In addition, I would get there like this, tired, I would get there feeling bad and, on the return, it is the same route, the same thing. Therefore, for me, it is very hard.” (Participant 6)

#### Health problems

Ten individuals reported difficulties in participating in the pulmonary rehabilitation program due to various health problems. Among these issues were clinical conditions that could have been caused by COVID-19, such as fatigue and dyspnea, and which could have been treated through the intervention that was declined.

Six individuals reported that pain influenced their decision not to participate in rehabilitation.

“It was because of the pain I feel. I feel pain, my dear, day and night.” (Participant 3)

Three individuals refused to participate due to being in the preoperative period.

“I have surgery scheduled.” (Participant 10)

Two individuals reported mobility difficulties, which hindered access to the rehabilitation center.

“For me, the most important factor is the difficulty I have due to my left leg. I use a cane, but even so, I have a great deal of trouble going out.” (Participant 17)

Nine individuals reported other health problems, including anemia, dyspnea, vertigo, fatigue, depression, heart disease, hypothyroidism, overactive bladder, and chikungunya.

“I cannot stand to take a bus; it bumps all the time. My bladder seems to be out of place, the doctor even said that my bladder is overactive.” (Participant 20)

“Because not only my leg hurts, but now I also feel breathless.” (Participant 6)

“I am going to have to do another test for hypothyroidism. I am truly discouraged.” (Participant 16)

#### Lack of time

To participate in the rehabilitation program, individuals were required to attend the university hospital three times a week for eight weeks. For this reason, seven individuals reported not having the time to attend the pulmonary rehabilitation program, primarily due to their work and domestic responsibilities.

Three individuals reported not having time due to work.

“It is a very long time, and takes almost half a day.” (Participant 20)

“I did not participate due to lack of time. When I left the hospital, I left feeling very well and went straight to work, and my job did not give me the time to do it.” (Participant 2)

Three individuals reported that a long commute to the rehabilitation center influenced their refusal to participate.

“I think it is more a matter of time. To stop being able to do this and to be able to go. Because it would be time spent on it.” (Participant 4)

Two individuals reported a lack of time due to household work.

“It takes time. In addition, there is no one to do things here at home.” (Participant 20)

#### No need perceived

Five individuals did not see the need to join a pulmonary rehabilitation program because they did not have any symptoms.

“I did not present any worsening in breathing. No problem about that.” (Participant 4)

### Minor themes associated with nonparticipation

Other themes related to nonparticipation in the pulmonary rehabilitation program were identified. Two individuals mentioned feeling demotivated, one needed more information about pulmonary rehabilitation, one expressed no interest in participating, and one cited a lack of medical encouragement.

“I received much information, but I did not want it anyway.” (Participant 5)

“I think we should have more information about the program because the basic health unit does not clarify this. In addition, for us to access these programs that you offer, we must go through the basic health unit, and they do not always give us the information we need.” (Participant 7)

“I am very demotivated to do things”. (Participant 13)

“I did not look into this because I did not have medical encouragement.” (Participant 8)

Although not reported as a reason for refusing to participate in the pulmonary rehabilitation program, ten individuals reported not receiving any information about the program. Another five individuals reported receiving but not remembering or knowing exactly how to describe the information.

“I did not receive any information.” (Participant 9)

“I received it but do not know how to report it to you. The girls explained it to me, but I do not know how to report it to you, I have difficulty remembering, and after COVID, I became forgetful.” (Participant 15)

## Discussion

This was the first qualitative study aiming to understand and discuss the barriers for individuals after COVID-19 hospitalization to participate in a pulmonary rehabilitation program. The themes related to refusal to participate were difficulties in accessing the rehabilitation center, health problems, lack of time, and not perceiving the need for rehabilitation.

Rehabilitation programs worldwide also reported difficulties in patient access to rehabilitation centers [16, 22]. Although the participants in our study had a relatively short median distance from their homes to the rehabilitation center (i.e., nine kilometers), commuting for more than 30 minutes was considered a barrier [23]. Additionally, access to rehabilitation is limited in several cities, and individuals often report distance and lack of transport as one of the main reasons for low acceptance or adherence [9, 22]. These factors should be considered when developing rehabilitation programs and require attention from entities responsible for social mobility to build rehabilitation centers near the community and in accessible places and provide a quality public transport system with reduced costs.

Health problems were reported as barriers to participation in the pulmonary rehabilitation program. Although symptoms related to post-COVID-19 syndrome (e.g., dyspnea and fatigue) were considered reasons for refusal, they should motivate participation due to the general improvements from rehabilitation [5–7]. Physicians and other multidisciplinary team members must be informed and prepared to treat health problems and comorbidities to prevent these barriers and allow the benefits of the pulmonary rehabilitation program for the individual. Additionally, alternative rehabilitation strategies should be considered to increase the participation and adherence of individuals with comorbidities and acute or chronic pain since walking, cycling, and performing resistance movements may be demotivating for this population. In this sense, pulmonary rehabilitation, such as aquatic exercise, should be considered since it is feasible and beneficial for individuals with mild to moderate chronic obstructive pulmonary disease [24].

Some individuals reported a lack of time as a barrier to participation. Work and household activities were previously reported by individuals with chronic lung diseases, corroborating the findings in our study [25]. Thus, alternative and innovative rehabilitation modalities may be established to overcome the lack of time and difficulties in accessing the rehabilitation center. For example, telerehabilitation has been adopted as a promising strategy for individuals with chronic respiratory diseases [26]. Home-based pulmonary rehabilitation may also be an alternative to overcome this barrier by performing low-cost and easy-to-implement exercises during free time [27]. Additionally, flexible schedules (including night shifts) may be a solution for individuals who work during the day and are interested in participating in the rehabilitation program.

Some individuals declined to participate in rehabilitation, as they did not perceive the need for it since they were feeling well. However, it is important to note that individuals with post-COVID-19 syndrome may not experience symptoms at rest, but they may appear during exercise [1, 6]. This perception could be attributed to the low physical activity levels in their daily routine.

Several individuals reported not receiving sufficient information on the pulmonary rehabilitation program. The lack of medical referrals and proper communication to emphasize the importance and benefits of rehabilitation after COVID-19 appeared to be decisive in some participants’ decision to take part in the rehabilitation program. In this sense, rehabilitation should be applied as a comprehensive intervention based on information and education, including actions allowing self-care, participation, and adherence to the treatment [8]. Additionally, educational actions for health professionals, family members, and caregivers are needed to increase awareness and knowledge of the benefits of pulmonary rehabilitation.

Our results provide important information about the barriers faced by individuals after COVID-19 hospitalization to participate in a pulmonary rehabilitation program. Additionally, they may have relevant impacts and raise questions for appropriate decisions to solve and overcome the barriers. Many barriers reported in this study can be addressed with appropriate methods of education and counseling for patients, such as addressing the lack of information about pulmonary rehabilitation. Thus, further studies need to evaluate the impact of solutions adopted to increase participation.

This study had some limitations. Individual assessments of the need for rehabilitation were not individually conducted. Nonetheless, all individuals were eligible due to severe COVID-19 requiring hospitalization. Individuals who agreed to participate in rehabilitation but did not adhere to the treatment were not included in this study. This decision was based on the insufficient sample size of individuals who agreed to participate in rehabilitation, and combining two different participant profiles into a single group could introduce an ’aggregation bias.’ Consequently, further studies are warranted to assess adherence to rehabilitation in this population. The sample comprised individuals from a specific social, economic, and cultural background, and therefore, caution should be exercised when interpreting the results for other populations. Nevertheless, the identified themes and subthemes were consistent with those in studies among individuals with other chronic respiratory diseases in various countries and contexts.

## Conclusion

Individuals who were hospitalized for COVID-19 presented several barriers to participating in a pulmonary rehabilitation program. These barriers included difficulties in accessing the rehabilitation center, health problems, lack of time, and the perception that rehabilitation was unnecessary. Therefore, measures to overcome these barriers are necessary to make the program available to a larger number of individuals and to convey the proven benefits of pulmonary rehabilitation to eligible patients.

## Supporting information

S1 File (DOCX)
