# Supplementary material for: Perceptions of individuals regarding barriers to participation in a pulmonary rehabilitation program after hospitalization due to COVID-19: A qualitative study
Source: PLoS One. 2023 Nov 28;18(11):e0294963. doi: 10.1371/journal.pone.0294963 (PMC10684017; doi:10.1371/journal.pone.0294963)
Supplement: S1 File — (DOCX) [file pone.0294963.s001.docx]

Perceptions of individuals regarding barriers to participation in a pulmonary rehabilitation program after hospitalization due to COVID-19: A qualitative study.

Rafaella Rabelo Polato, Cristino Carneiro Oliveira, Yuri Augusto de Souza Miranda, Leandro Ferracini Cabral, Carla Malaguti, Anderson José

**Transcription of the interviews**

**Participant 1**

1. **Why do you think you were referred to a pulmonary rehabilitation program?**

Response: Because of Covid...

1. **I understand that you chose not to participate in the pulmonary rehabilitation program. Can you tell me about it?**

Response: So at the time, I... I didn't, actually, choose not to participate. I had a health issue related to the gallbladder, so I had to have surgery. I took a long time to find out that I had a problem with my gallbladder, spent a long time seeking medical help, and after I had the surgery, I couldn't find time to do it...

1. **What was the most important factor that prevented you from participating in the pulmonary rehabilitation program?**

Response: It was this illness I went through... with the gallbladder.

1. **What information did you receive about the pulmonary rehabilitation program?**

Response: Some... I just don't remember (laughs) it's been a long time...

1. **How do you think a pulmonary rehabilitation program is?**

Response: I have no idea... I think it's similar to what you did at home, training breathing... I think...

1. **Do you think that the rehabilitation would provide any benefit for your health?**

Response: For me, yes, because I have a lot of respiratory problems, I have rhinitis, sinusitis, so I always have rhinitis crises, and I'm always short of breath...

1. **Do you consider participating in a pulmonary rehabilitation program in the future?**

Response: Yes...

1. **In the future, for you to participate in a rehabilitation program, what circumstances should change?**

Response: For me, the problem is that it would be at the university hospital. It would be too far away. And I would need my husband to be available to take me... understand? And that's just the issue of it being far from here.

**Participant 2**

- 1. **Why do you think you were referred to a pulmonary rehabilitation program?**

Response: Due to my COVID condition, since I was hospitalized for six days, I believe it's related to some pulmonary issue that pulmonary physiotherapy could have helped me with.

- 1. **I understand that you chose not to participate in the pulmonary rehabilitation program. Can you tell me about it?**

Response: I did not participate due to lack of time. When I left the hospital, I left feeling very well and went straight to work, and my job did not give me the time to do it.

- 1. **What was the most important factor that prevented you from participating in the pulmonary rehabilitation program?**

Response: Work responsibilities.

- 1. **What information did you receive about the pulmonary rehabilitation program?**

Response: I received information when I was in the hospital, and later, Unimed Pleno contacted me to provide information, but they didn't provide details about what the rehabilitation would actually entail.

- 1. **How do you think a pulmonary rehabilitation program is?**

Response: I believe it involves specific breathing exercises.

- 1. **Do you think that the rehabilitation would provide any benefit for your health?**

Response: Yes, definitely.

- 1. **Do you consider participating in a pulmonary rehabilitation program in the future?**

Response: If I need to, I think I would.

**8) In the future, for you to participate in a rehabilitation program, what circumstances should change?**

Response: My schedule.

**Participant 3**

1. **Why do you think you were referred to a pulmonary rehabilitation program?**

Response: I had a lot of leg pain... I feel a lot of body pain... my legs don't work well, I can't stand for long.

**2)** **I understand that you chose not to participate in the pulmonary rehabilitation program. Can you tell me about it?**

Response: Because of the difficulty in taking me there. I want to do it, but it needs to be in a closer place.

**3) What was the most important factor that prevented you from participating in the pulmonary rehabilitation program?**

Response: It was because of the pain I feel... I feel pain all day and night. During the day, it's manageable, but at night, I can only sleep with the help of medication.

**4)What information did you receive about the pulmonary rehabilitation program?**

Response: I don't know how to answer that...

**5) How do you think a pulmonary rehabilitation program is?**

Response: I think so... that's why I want to do it.

**6) Do you think that the rehabilitation would provide any benefit for your health?**

Response: I think so... that's why I want to do it.

**7) Do you consider participating in a pulmonary rehabilitation program in the future?**

Response: I want to...

**8) In the future, for you to participate in a rehabilitation program, what circumstances should change?**

Response: Change? I don't know... I don't know how to answer that...

**Participant 4**

1. **Why do you think you were referred to a pulmonary rehabilitation program?**

Response: So, I believe it's because I had COVID, and because of that, it affected my lungs, and... some procedure should be done to improve the condition of the organ.

**2) I understand that you chose not to participate in the pulmonary rehabilitation program. Can you tell me about it?**

Response: Yes, so I chose not to participate because I had follow-up appointments after I was discharged. I had follow-ups with doctors, went to the pulmonologist. I did not present any worsening in breathing. No problem about that. I consulted with the local doctor and the pulmonologist, and they said everything was fine. So, I didn't worry about seeking any rehabilitation because I truly believe I didn't have any lingering effects.

**3) What was the most important factor that prevented you from participating in the pulmonary rehabilitation program?**

Response: Well, it didn't exactly prevent me, but what made me not seek it was precisely that - the fact that I had consultations with doctors, and there were no problems or difficulties in breathing. Seeing that everything was indeed okay, I didn't worry about it.

**4) What information did you receive about the pulmonary rehabilitation program?**

Response: That it would involve exercises... to improve respiratory condition, breathing exercises to try to enhance respiratory condition.

**5) How do you think a pulmonary rehabilitation program is?**

Response: So, I imagine it involves... exercises, respiratory exercises, to improve the muscle strength of the organ in that sense.

**6) Do you think that the rehabilitation would provide any benefit for your health?**

Response: Yes, definitely. I believe it would improve breathing, enhance... help with physical conditioning, for example, being able to do an activity and breathe better, achieve that kind of thing.

**7) Do you consider participating in a pulmonary rehabilitation program in the future?**

Response: Well, I haven't really... never thought about it, never... it's not something I've actively considered, like 'Oh, I'd like to do that.' But if it were offered, if I had the means to do it, if it were paid for, for instance, if I had the time, I would do it.

**8) In the future, for you to participate in a rehabilitation program, what circumstances should change?**

Response: I think it is more of a matter of time. To stop being able to do this and to be able to go. Because it would be time spent on it. And if it were paid, financial resources as well.

**Participant 5**

**1) Why do you think you were referred to a pulmonary rehabilitation program?**

Response: Because of the Covid I had.

**2) I understand that you chose not to participate in the pulmonary rehabilitation program. Can you tell me about it?**

Response: Lack of patience and willingness.

**3) What was the most important factor that prevented you from participating in the pulmonary rehabilitation program?**

Response: "The same thing... lack of willingness, lack of patience..."

**4) What information did you receive about the pulmonary rehabilitation program?**

Response: I received much information, but I did not want it anyway.

**5)How do you think a pulmonary rehabilitation program is?**

Response: "I don't know..."

**6) Do you think that the rehabilitation would provide any benefit for your health?**

Response: "I don't know either..."

**7) Do you consider participating in a pulmonary rehabilitation program in the future?**

Response: No.

- 1. **In the future, for you to participate in a rehabilitation program, what circumstances should change?** Response: I would need to have a lot of patience; it's very difficult for me to participate in something like that.

**Participant 6**

**1) Why do you think you were referred to a pulmonary rehabilitation program?**

Response: Because I was hospitalized last year, in September, for 19 days at University Hospital, because of my leg, which was getting stiff. The doctors rushed me there, and I was hospitalized because they were afraid it was a thrombosis. I had tests done there, but they couldn't detect what it was. Until now, it's undiagnosed; they say it's thrombosis, but nothing is confirmed. After I was discharged, I spent 19 days doing physiotherapy there, you understand? So, I received the referral to go to University Hospital to start physiotherapy. I called them after reaching home and started quickly. They called me quickly, and I was received very well. After that, I stopped because the pain became intense. I started again when I realized I needed it. Renata (the physiotherapist), called me. I have her on WhatsApp, and we chatted there. She said, 'Eva, you can't give up because if you stay inactive, it will get worse.' So, I started going again. I get up early in the morning because the bus comes very early here, and I go, you understand? Every Wednesday, and later we changed to Fridays. I haven't used the treadmill there yet; I recently started cycling there as Renata put me on the stationary bike. She was initially hesitant about putting me on the treadmill because of my heart problem, but I started on the bike and those leg-stretching exercises. My visit to University |Hospital is that day for me when I leave in the morning, from 8:00 to 9:00, and then I wait for the bus. Afterward, there's another bus to catch. In summary, I arrive home at 12:00. That day is over for me; I don't do much at home. It's just pain, pain. I know exercising causes pain, so it's a lot of pain. I elevate my legs, and the emotional pain goes away. I already have depression issues, and this worsened it. My psychiatrist mentioned it, and I'm doing online therapy, which I was already doing. Now they... they... I don't know why, maybe because they found out I had COVID, so I've been doing online therapy for about three months. Every Monday at 3:00, I have a session with the physiotherapist. Some days, the pain is so much that we do the session on the bed. I tell her, 'Today, I can't get up.' You understand? That's it.

**2) I understand that you chose not to participate in the pulmonary rehabilitation program. Can you tell me about it?**

Response: I think it's because of my physical condition, having to go back there. I was afraid of it being another day that didn't match, you know? For me to do both, do you understand? Because now, it's affecting me. Because not only my leg hurts, but now I also feel breathless. The cardiologist said that this shortness of breath is not related to the heart; it's related to my lungs. The cardiologist requested a CT scan, and on Friday, I will go to the clinic to have it done.

**3) What was the most important factor that prevented you from participating in the pulmonary rehabilitation program?**

Response: My... my comorbidity... for me to go up there.

**4)** **What information did you receive about the pulmonary rehabilitation program?**

Response: Um... none...

**5) How do you think a pulmonary rehabilitation program is?**

Response: I can't even imagine...

**6) Do you think that the rehabilitation would provide any benefit for your health?**

Response: I think so... I think so... It will improve fatigue, you know? If you walk a little, I get breathless, you understand? I think so...

**7) Do you consider participating in a pulmonary rehabilitation program in the future?**

Response: Yes...

**8) In the future, for you to participate in a rehabilitation program, what circumstances should change?**

Response: I don't know about the... there to tell you about this; I don't know. I haven't started yet. Now, like I did physiotherapy, I can talk about it, I know it... I dealt with it there. So, I have a lot of difficulty with mobility, you understand? And getting to University Hospitl, when I say this, I do physiotherapy at University Hospital, and everyone looks at me and says, 'Why so far?' Because I was hospitalized there at the hospital, and they referred me there. I like it there because they check my glucose, blood pressure, and everything. In my case, I can't do just any physiotherapy because I have a heart problem, you understand? And I have to leave my house in the morning, like, early in the morning, around five in the morning, I catch a bus right at my doorstep, so that part is easy-going. But when I get downtown, I have to hurry because there's another one that comes at that time, or else I won't make it to Renata's (the physiotherapist) place. You see, everyone has their own schedule, and I can't go over someone else's time. So, I have to walk fast to catch the next bus, and that bus is usually packed. There have been times when the bus... broke down, and I had to call Renata and say, 'Renata, I can't make it, the bus broke down, you know?' I'm heading back home, you understand? There's no way. The bus from here to there rumbles and jumps and only God knows what else. In addition, I would get there like this, tired, I would get there feeling bad and, on the return, it is the same route, the same thing. Therefore, for me, it is very hard.

**Participant 7**

**1) Why do you think you were referred to a pulmonary rehabilitation program?**

Response: Because at the time I had an exam, the doctor said that I needed to come back for follow-up to see how I was after having had Covid. But I didn't return due to the paperwork I had to submit for surgeries.

**2) I understand that you chose not to participate in the pulmonary rehabilitation program. Can you tell me about it?**

Response: Well, due to the surgery I had to undergo, I was concerned about doing other tests. I was feeling a little better, not completely recovered, but a bit better to be able to do the other tests and try to undergo the other surgeries that, in the doctor's opinion, were important for me to have.

**3) What was the most important factor that prevented you from participating in the pulmonary rehabilitation program?**

Response: Well, due to the pandemic, it was very difficult to access this kind of service, especially through the doctor. It became more complicated to have access to these services at that time.

**4) What information did you receive about the pulmonary rehabilitation program?**

Response: I didn't receive any information.

**5) How do you think a pulmonary rehabilitation program is?**

Response: I have no idea what it's like.

**6) Do you think that the rehabilitation would provide any benefit for your health?**

Response: Yes, I think so, because I had some... well, let's say, after-effects of Covid. I was one Adriana before, and I became a different Adriana after. I felt the difference in my health after Covid.

**7) Do you consider participating in a pulmonary rehabilitation program in the future?**

Response: I would like to participate, yes, I would.

**8) In the future, for you to participate in a rehabilitation program, what circumstances should change?**

Response: I think we should have more information about the program because the basic health unit does not clarify this. In addition, for us to access these programs that you offer, we must go through the basic health unit, and they do not always give us the information we need.

**Participant 8**

**1) Why do you think you were referred to a pulmonary rehabilitation program?**

Response: I think it's necessary when a person has symptoms; they should be referred because... it can cause... more serious problems down the line. Several people have developed lung problems after having COVID. In my case, I didn't need it; I didn't feel anything. Well, at the very beginning when I hadn't discovered any lung involvement or anything, I was picking up my aunt because she was feeling unwell. She's elderly, 95 years old, not 85, excuse me. I went down the stairs with her, down the hill, put her in the car. When I... put her in the car and stood up, I thought, 'What's happening?' That shortness of breath, fatigue... and I couldn't even go back up the hill properly. It was right at the beginning of COVID; I hadn't even felt unwell enough to go to the hospital. I felt sick at home; my oxygen saturation was 55%. After that day, my wife helped me because I was almost fainting in the bathroom. That's when I turned green, and I went straight to the hospital and was hospitalized for 12 days. If I'm not mistaken, it was one or two days after.

**2) I understand that you chose not to participate in the pulmonary rehabilitation program. Can you tell me about it?**

Response: I had the complete follow-up with the pulmonologist. She followed me every two months when I left the hospital, a month later, and then two months later. So, I had a CT scan, and my lungs recovered very well. So I didn't feel any sequelae, didn't need it, resumed sports without any problem.

**3) What was the most important factor that prevented you from participating in the pulmonary rehabilitation program?**

Response: I didn't have any lung sequelae, no shortness of breath, nothing at all.

**4) What information did you receive about the pulmonary rehabilitation program?**

Response: I didn't delve into it because I didn't need to. So, I did not deepen into it I did not look into this because I did not have medical encouragement.

**5) How do you think a pulmonary rehabilitation program is?**

Response: Well... It's hard to say because I didn't need it. But I think it would be interesting for those who need it because they follow the patient at different intervals to monitor progress. If you don't follow up with those who really needed this monitoring, it might cause a more significant problem later.

**6) Do you think that the rehabilitation would provide any benefit for your health?**

Response: In my case, not now because I have no problems with... breathing. I do everything without getting tired, absolutely nothing, nothing, nothing in my lungs. In my specific case, no. But I know people, including my father, for whom it would still be beneficial because he still feels something.

**7) Do you consider participating in a pulmonary rehabilitation program in the future?**

Response: In the future, if I need to, yes. No problem.

**8) In the future, for you to participate in a rehabilitation program, what circumstances should change?**

Response: Ah, if it interfered with my daily life, if I couldn't do my activities, I... would definitely seek it out.

**Participant 9**

**1) Why do you think you were referred to a pulmonary rehabilitation program?**

Response: So... I don't know. I was admitted to University Hospital Santa Catarina for breast surgery, and when I got there, they did a rapid test and said I had COVID, but the doctor didn't let me go because I wasn't feeling anything.

**2) I understand that you chose not to participate in the pulmonary rehabilitation program. Can you tell me about it?**

Response: The reason... Well, because it's a bit out of the way. I live in Matias, and you have to keep going to the city and University Hospital every day, and it costs me. Also, the public service takes time to respond... release the money after a medical examination.

**3) What was the most important factor that prevented you from participating in the pulmonary rehabilitation program?**

Response: The most important factor? Ah... Leaving the spot for someone who really needs it, right!?

**4) What information did you receive about the pulmonary rehabilitation program?**

Response: I did not receive any information.

**5) How do you think a pulmonary rehabilitation program is?**

Response: Ah... It might, but I don't think I need it.

**6) Do you think that the rehabilitation would provide any benefit for your health?**

Response: Ah... It might, but I don't think I need it.

**7) Do you consider participating in a pulmonary rehabilitation program in the future?**

Response: Ah, maybe yes in the future.

**8) In the future, for you to participate in a rehabilitation program, what circumstances should change?**

Response: What circumstances? Ah... Financially, mainly... The issue of getting around, paying for transportation, and also time because in the future, I'll be going back to work.

**Participant 10**

**1) Why do you think you were referred to a pulmonary rehabilitation program?**

Response: Ah... I don't even remember the day... I don't remember.

**2) I understand that you chose not to participate in the pulmonary rehabilitation program. Can you tell me about it?**

Response: It's because I was mostly at home, I was just at home.

**3) What was the most important factor that prevented you from participating in the pulmonary rehabilitation program?**

Response: The problem is going, I can't go... It's difficult.

**4) What information did you receive about the pulmonary rehabilitation program?**

Response: None... None.

**5) How do you think a pulmonary rehabilitation program is?**

Response: Well, it's... it's an improvement, right... Checking how the lungs are, right... Checking how the lungs are... I remember that day when they took me to do the exam... I remember.

**6) Do you think that the rehabilitation would provide any benefit for your health?**

Response: I don't know... I think so, right.

**7) Do you consider participating in a pulmonary rehabilitation program in the future?**

Response: Not now, right?

**8) In the future, for you to participate in a rehabilitation program, what circumstances should change?**

Response: It's because... From what I see, I can't go, it's difficult... It's difficult to get there... And I... I have surgery scheduled., I don't know when it will be. Tomorrow, I'll go to the doctor to see how my liver is reacting, if there's anyone there to do it.

**Participant 11**

**1) Why do you think you were referred to a pulmonary rehabilitation program?**

Response: I think... I don't even know why. Because sometimes the doctor thought I had something in my lungs because of a cough I had, but my cough was already from COVID. My throat was very irritated, but he said... At University Hospital, they didn't have a device to do the test, I had to do it privately, but he also didn't tell me if I should go here, there, or somewhere else.

**2) I understand that you chose not to participate in the pulmonary rehabilitation program. Can you tell me about it?**

Response: Because I am fine, I don't feel anything, no back pain... Now he mentioned the anemia, when I climbed the hill to go to the market I felt a lot of pain... Here... So I said, the next time, now, when I go to the doctor I will talk to him, but he told me to do the tests and it showed that I am... Because anemia also causes leg pain.

**3) What was the most important factor that prevented you from participating in the pulmonary rehabilitation program?**

Response: To get there... It's difficult, right? He works, the other one works, and for me to pay for a car to go there and back is quite a bit... And I'm spending a lot on medicines... I've already spent and now... And now I'm spending on these, so we'll see.

**4) What information did you receive about the pulmonary rehabilitation program?**

Response: None.

**5) How do you think a pulmonary rehabilitation program is?**

Response: Ah... I don't know because... I think... This lung thing, I think I don't feel anything, because... Because the doctor removed it, but I don't even know what happened or anything, he didn't say anything.

**6) Do you think that the rehabilitation would provide any benefit for your health?**

Response: Now I don't know, because I only see it from the outside.

**7) Do you consider participating in a pulmonary rehabilitation program in the future?**

Response: If necessary, right...

**8) In the future, for you to participate in a rehabilitation program, what circumstances should change?**

Response: I can't say. I think if it were urgent and all... But, thank God... The distance, it's very far there.

**Participant 12**

1. **Why do you think you were referred to a pulmonary rehabilitation program?**

Response: Well, I was... hospitalized, with COVID symptoms and stayed in the hospital for 3 months, with COVID, undergoing treatment there, until I could continue treatment at home.

**2) I understand that you chose not to participate in the pulmonary rehabilitation program. Can you tell me about it?**

Response: No, actually, I would really like to participate. I just won't be able to due to the schedule... Because I work from 8 am to 6 pm, I only have... an hour for lunch, so... It's very difficult for me, right, to leave here from the city center and go to University Hospital for rehabilitation... It would be because of the schedule, if it were later, if it could be extended, I would like to do it.

**3) What was the most important factor that prevented you from participating in the pulmonary rehabilitation program?**

Response: Actually, the schedule.

**4) What information did you receive about the pulmonary rehabilitation program?**

Response: So, I was informed that there would be this rehabilitation for those who had COVID, to... to delay, to improve breathing... Those things... Something like that... It wouldn't be for a long time, but it would be in this idea, to get back to... to working with the lungs, to really improve breathing. On the day I stayed, later... I have asthma, and after a while, after COVID, I started using the inhaler again, so every day I use the inhaler again because of COVID.

**5) How do you think a pulmonary rehabilitation program is?**

Response: I believe it's what I explained, I don't know the details and specifics, but unfortunately, it's... It's just because of this schedule that I won't be able to do it.

**6) Do you think that the rehabilitation would provide any benefit for your health?**

Response: I believe so. I believe it would help me a lot because there really is difficulty, fatigue, you know? It's... greater after this, various factors that remained as symptoms even after COVID that I still feel.

**7) Do you consider participating in a pulmonary rehabilitation program in the future?**

Response: Definitely.

**8) In the future, for you to participate in a rehabilitation program, what circumstances should change?**

Response: Well, if there were a schedule... In the evening, right? If there was... An... Right... That passed by, at least until... I don't know, eight, nine o'clock, like that... So that other working people could also do it, that would be ideal, you know?

**Participant 13**

**1) Why do you think you were referred to a pulmonary rehabilitation program?**

Response: Did not answer.

**2) I understand that you chose not to participate in the pulmonary rehabilitation program. Can you tell me about it?**

Response: Well... it's very difficult for me to go... to accept... it's quite difficult... It is too far; I need a taxi. It would be difficult because of the commute.

**3) What was the most important factor that prevented you from participating in the pulmonary rehabilitation program?**

Response: Ah... everything, the exams... those things, right? I am very demotivated to do things.

**4) What information did you receive about the pulmonary rehabilitation program?**

Response: None.

**5) How do you think a pulmonary rehabilitation program is?**

Response: Well, I don't know... I don't know what to say about it... They only mentioned it when I arrived... they said I had pneumonia and a bit of wheezing... They put me on a stretcher... On a drip... On oxygen... On the first day I was there. But I don't know what they do in the lungs, I should know, but I was feeling... tired, all these things... And I had a bit of water in my lungs... They mentioned that, that I had a bit of water in my lungs... I was feeling tired, walking a little... I raised my hand and got tired... Before I knew it, I was getting tired. Now here, like this, I'm feeling... Sometimes when I go out a bit... a bit far... When I start to climb the ramp of the building here... I start to get tired.

**6) Do you think that the rehabilitation would provide any benefit for your health?**

Response: I think so.

**7) Do you consider participating in a pulmonary rehabilitation program in the future?**

Response: Ah... Yes, what do you mean? For physiotherapy? To get better? Ah... So, I wanted to do that.

**8) In the future, for you to participate in a rehabilitation program, what circumstances should change?**

Response: Ah... For me to go there and participate? Ah... I don't know... It's very far, I need a taxi.

**Participant 14**

**1) Why do you think you were referred to a pulmonary rehabilitation program?**

Response: I felt very tired afterwards, any minimal effort I made, even the usual household chores, made me feel very tired. It's like I felt my body heavy, legs heavy, and respiratory fatigue, as if I had done a very intense workout with the small things I did.

**2) I understand that you chose not to participate in the pulmonary rehabilitation program. Can you tell me about it?**

Response: Yes, actually, it's because of the travel, you see. I have a young child, so it would be complicated. I would need someone available to take care of him, and I was already in need of help from family for both him and me because I couldn't do many things. There wouldn't be the availability of someone to take care of him since they were already helping me. It's complicated for us to keep asking for help all the time, especially since I was already demanding a lot from my family. And about the distance, it wouldn't be that much of an issue for me, but I wouldn't be at home, so I would have to take my child to stay with someone, come back... and then there's the cost, for fuel, the time for transportation. The University Hospital is located in a place where public transport is not very convenient, so it would take more time if I were to take the bus.

**3) What was the most important factor that prevented you from participating in the pulmonary rehabilitation program?**

Response: I think it's the distance.

**4) What information did you receive about the pulmonary rehabilitation program?**

Response: None.

1. **How do you think a pulmonary rehabilitation program is?**

Response: I think it involves conditioning, respiratory exercises using those blowing devices, and stretching because we tend to have a bad posture from lying down a lot. I was lying down a lot at home and then in the hospital too. Being isolated in a room affects your posture; you lose strength and end up lying down more due to the disease. I felt pain all over my body from sitting or lying down and having to stay isolated. You don't have a proper position, and it's the same in the hospital because you're confined to the room and bed, restricted in movement, constantly on the bed, I was practically almost 14 days in a bed, so I think it's due to that too.

**6) Do you think that the rehabilitation would provide any benefit for your health?**

Response: Definitely... the possibility of returning to normal life because even with a short distance or a little uphill, which I used to climb, I would get very tired. I believe I would have recovered in a shorter time, felt shortness of breath, a lot of fatigue.

**7) Do you consider participating in a pulmonary rehabilitation program in the future?**

Response: Yes, definitely.

**8) In the future, for you to participate in a rehabilitation program, what circumstances should change?**

Response: I think it would have to be in a more central location. I think the location, despite the structure being very good at University Hospital, is a factor that greatly hinders things in general. Also, during that time, I was on sick leave, and these schedule adjustments are quite complicated. I believe that many people might not have been able to participate because of work schedules, fear of losing their job or facing some sort of punishment for taking time off for health treatment, even though it's a medical treatment, we know these things happen.

**Participant 15**

**1) Why do you think you were referred to a pulmonary rehabilitation program?**

Response: I went to University Hospital Santa Terezinha to see my gastrologist because I have a heart problem. I told the doctor that I was very tired and had a lot of shortness of breath. So, she said she would put me in the physiotherapy at HU. I went to the cardiologist, and it was prescribed because of the fatigue, shortness of breath, and body aches that I felt, you know?

**2) I understand that you chose not to participate in the pulmonary rehabilitation program. Can you tell me about it?**

Response: So, I started going, but it was difficult for me. I was going, but there was no one to go with me. I had a lot of dizziness, and I also had the aftermath of feeling very dizzy during physiotherapy. I was feeling very dizzy on the street, due to pressure and labyrinthitis. I didn't have company because I already had a lot of doctors to see, and I couldn't ask someone to go with me for physiotherapy because I already had to arrange someone to accompany me to the doctors. My legs were very weak, you know? I felt very bad about having to take two buses. I didn't pay the fare, that wasn't a problem, but taking two buses, you know? I felt very itchy, and I arrived very tired. It was helping me, but it was also harming me. I was getting very tired.

**3) What was the most important factor that prevented you from participating in the pulmonary rehabilitation program?**

Response: I think it was because... I was much better with physiotherapy. It was about five times a week or maybe more, I think it was about three or four times. My legs were weak, and I had dizziness. Also, before I had COVID, I already felt shortness of breath. After having COVID, it got worse, but thanks to God and our lady, now after physiotherapy, I've improved 90%.

**4) What information did you receive about the pulmonary rehabilitation program?**

Response: I received it but do not know how to report it to you. The girls explained it to me, but I do not know how to report it to you, I have difficulty remembering, and after COVID, I became forgetful.

**5) How do you think a pulmonary rehabilitation program is?**

Response: I don't know... they explained it to me, but I don't know how to explain it, and I don't know for myself either.

**6) Do you think that the rehabilitation would provide any benefit for your health?**

Response: It would, because it helped me a lot, it helped me a lot.

**7) Do you consider participating in a pulmonary rehabilitation program in the future?**

Response: I would.

**8) In the future, for you to participate in a rehabilitation program, what circumstances should change?**

Response: I would need someone to go with me, someone to accompany me.

**Participant 16**

**1) Why do you think you were referred to a pulmonary rehabilitation program?**

Response: Ah... I'm still the same, I don't know if... if... Well... If it's something like... What I have is hypothyroidism and, after COVID, I had a sequela of joint pain, my face hasn't come back yet... It hasn't come back yet... Like... I'm very forgetful, you know? And... Also, I don't know if it's due to hypothyroidism; the thyroid test I did was... last month, it was 13.30 and then it went down to 12.6, and then I'm walking very discouraged, really, very discouraged. Like... How to say? I'm not connecting much... I'm very... I'm like this... There are times when I want to say something and I forget, it slips from my mind, you know? I have joint pain, I feel that a lot. After I had COVID, I had an MRI of the hip, and both are debilitated, and I think that's why they referred me to physiotherapy. So, I have a lot of pain in the femur, in the hip area, so the doctor referred me because of this joint pain.

**2) I understand that you chose not to participate in the pulmonary rehabilitation program. Can you tell me about it?**

Response: Girl, wow... I was talking to my sister. I am going to have to do another test for hypothyroidism. I am truly discouraged. It's because of that, I'm... like... very unmotivated, you know? But really, very unmotivated.

**3) What was the most important factor that prevented you from participating in the pulmonary rehabilitation program?**

Response: It's precisely this lack of motivation; the bus stops here, gets off here, very close... I also don't pay for the bus because I'm almost 78 years old... So, I don't pay for the bus, and also... It would be more about the lack of motivation.

**4) What information did you receive about the pulmonary rehabilitation program?**

Response: None.

1. **How do you think a pulmonary rehabilitation program is?**

Response: Honestly, I don't know... Rafaella, what I did was like... The lady... told me to walk, like, on the stripes... All I remember was that... Also, my cholesterol was high at the time, it was 302, and my blood pressure also goes up if I get tense there.

**6) Do you think that the rehabilitation would provide any benefit for your health?**

Response: I think so because... I have to exercise, I have to reanimate... My goodness... yesterday my sister was talking to me... Also Rafaella... It's from University Hospital Dom Bosco, right? My sister was even talking to me, because I lost... I lost almost 12 kg after this COVID thing, but I have a lot of breast tissue, I even... They gave me the referral to do breast reduction, but at the time I was also unmotivated and didn't want to. So, my sister said that... She knows Larissa at HU, do you know Larissa there? My goodness, since my sister had breast reduction surgery, could you talk to her for me, because it's bothering me a lot, because I have a herniated disc problem, and it's weighing down, you know? Because I'm losing weight, but it continues. I'm Darci's sister, my sister had surgery with her.

**7) Do you consider participating in a pulmonary rehabilitation program in the future?**

Response: I would, because I don't know why the doctor who referred me... I do wish, yes.

**8) In the future, for you to participate in a rehabilitation program, what circumstances should change?**

Response: It's because for me, I prefer these morning appointments, and at that time, I think they only had it in the afternoon, the lady told me.

**Participant 17**

**1) Why do you think you were referred to a pulmonary rehabilitation program?**

Response: Because I had COVID, right!? And I already have bronchitis, right!? I already use... I use an inhaler, even outside of COVID, right!? For this... For this reason, I was referred. When I had COVID, I felt... I felt it in my breathing, right!? A bit of fatigue... Quite tired, right!? More difficulty walking... Moving around. Apart from the issues I already have, right!? I had... I had arthroplasty, had a prosthesis placed in my left leg, right!? I have difficulty... For example, getting on a bus, getting off... I usually do it when... When needed, but, it's very difficult for me.

**2) I understand that you chose not to participate in the pulmonary rehabilitation program. Can you tell me about it?**

Response: Exactly, right!? Because... Because of the distance, right!? Because for me... I live in Santa Terezinha, I would have to take two buses, right!? So, the bad thing is that the buses don't always stop for us... Near the sidewalk, so sometimes it's difficult to get on the bus, sometimes difficult, too, to get off, so I... I preferred not to participate.

**3) What was the most important factor that prevented you from participating in the pulmonary rehabilitation program?**

Response: Yeah. For me, the most important factor is the difficulty I have due to my left leg. I use a cane, but even so, I have a great deal of trouble going out.

**4) What information did you receive about the pulmonary rehabilitation program?**

Response: I was informed that it would be to do... Pulmonary physiotherapy, right!? To help with breathing, right? Which in my case would even be good for bronchitis, right!? I need it, whenever I have a crisis I already... I already do... I have to do it, sometimes I have to go to... To the clinic to do it when the physio is available. I received information that I should go, but due to the difficulties, I couldn't.

**5) How do you think a pulmonary rehabilitation program is?**

Response: How do I think? I... Well... I imagine, because a few years ago I had a lot of difficulty when I started mine, this... The bronchitis, I had to do pulmonary physiotherapy, so I imagine it would be like that,

right!? Some exercises, right!? They gave us a device there to blow into a ball, right!? Also, for example, cover one nostril, breathe... I know I've done this kind of treatment when I had the onset of asthma, so I imagine it would be like that.

**6) Do you think that the rehabilitation would provide any benefit for your health?**

Response: Yes, I think it would.

**7) Do you consider participating in a pulmonary rehabilitation program in the future?**

Response: I do consider.

**8) In the future, for you to participate in a rehabilitation program, what circumstances should change?**

Response: The transportation, right!? Yeah, the transportation, right!? For example, for this purpose, there's no support car, right!? There is no car to take me, and to pay for a vehicle, an Uber or taxi, is very expensive. To do this more often.

**Participant 18**

1. **Why do you think you were referred to a pulmonary rehabilitation program?**

Response: Ah... Rehabilitation, I... I... Could go, but I had, uh... Someone doing a research and I accompanied, this research was as follows... We inhale through the nose for 5 minutes, then exhale, then it goes to the lungs... Then the most difficult she gave me was breathing for 10 seconds, then... Then it gets more difficult, so I tried, but I couldn't hold on until the 10 seconds and exhaled. That's the lung exercise. There was no change, a doctor came here because I have a plan with Unimed, right!? So, a doctor came here one day, and she looked at everything in me, lungs, heart, and... Yes... This doctor from Unimed who was

here, she did all the tests on me, so... She told me to do the breathing exercise for the lungs, but I wasn't feeling anything, she looked at everything in me and said everything was fine.

**2) I understand that you chose not to participate in the pulmonary rehabilitation program. Can you tell me about it?**

Response: That's it... That's it... It's because I depend on someone to take me, then it's not possible... Because Mauricéia (daughter) has her problems there with her husband who had this, right!? Marilene works, but if I ask after she comes from work... she does take me... But it's complicated.

**3) What was the most important factor that prevented you from participating in the pulmonary rehabilitation program?**

Response: This issue of having someone to take... Especially because you mentioned needing a companion... So... I have this difficulty.

**4)** **What information did you receive about the pulmonary rehabilitation program?**

Response: They've already explained to me.

**5) How do you think a pulmonary rehabilitation program is?**

Response: I think it's very good. Ahh... This breathing that goes to the lungs and then releases the air. That helps a lot for the lungs.

**6) Do you think that the rehabilitation would provide any benefit for your health?**

Response: It helps because... After that, every now and then I do it, because... It's important, as the person said. It's important because... You inhale and then exhale. It's important for the lungs.

**7) Do you consider participating in a pulmonary rehabilitation program in the future?**

Response: Yes, I can even participate, if someone takes me.

**8) In the future, for you to participate in a rehabilitation program, what circumstances should change?**

Response: What would it take? It... It depends, right!? Someone to take me.

**Participant 19**

**1) Why do you think you were referred to a pulmonary rehabilitation program?**

Response: Why do I think? It's because I went to the doctor, right!? I had COVID... So, in this case... Right!? Like, sometimes, the way we deal with fatigue changes a little, right!? I felt a lot of fatigue, so he even, like... He mentioned that I could go for pulmonary rehabilitation, right!? But like... It's not possible, it's not possible.

1. **I understand that you chose not to participate in the pulmonary rehabilitation program. Can you tell me about it?**

Response: I couldn't participate because I work, right!? I'm at work all day, the timing doesn't work for me, it's just not possible, and... it's far, right!? Transportation and all. I would have to take four buses, two to go and two to return. In addition, the ticket, four tickets would be a bit expensive. And timewise, it just doesn't work.

3) What was the most important factor that prevented you from participating in the pulmonary rehabilitation program?

**Response: Yeah... The fare, 4 fares would be a bit much.**

**4) What information did you receive about the pulmonary rehabilitation program?**

Response: Very little... Very little.

1. **How do you think a pulmonary rehabilitation program is?**

Response: I think it involves some exercises, right!? I'm not sure how, but it's an exercise that is given to the person to improve shortness of breath... Breathing... I think that's it.

**6) Do you think that the rehabilitation would provide any benefit for your health?**

Response: Yes, I think so

**7) Do you consider participating in a pulmonary rehabilitation program in the future?**

Response: Oh, in the future, yes, for sure.

**8) In the future, for you to participate in a rehabilitation program, what circumstances should change?**

Response: The program timings... Could be like this... I know it's kind of difficult, but a bit earlier and a bit later. Because maybe... Let's say... They could add more staff, right!? And in the afternoon, let's say, at 6 pm perhaps... I know it's not... But for people who don't have time, have these kinds of timings. The fare also becomes too much if you have to do it every day, it's too much. I think there should be... If people who go... The municipality should provide a free pass, right, for people who can't afford it. Because my nephew, when he had attention deficit treatment... The municipality provided a pass for him and someone to take him... For the companion, because nowadays no one can afford to pay for transportation.

**Participant 20**

**1) Why do you think you were referred to a pulmonary rehabilitation program?**

Response: I felt tired... Tired... And I went to one of the doctors... Several doctors... I had an X-ray and they said there was nothing, but that, like this, a spot remained, which they said was pneumonia, and I felt very cold, always feeling cold... and like this... I changed, it's like everything in me changed after I was intubated... Nothing is the same as before... Nothing. I think everything changed... Like... To walk... Eating, no... And another thing that I... The doctor couldn't even explain what it was... It's not medication... I'm sleeping too much, I have a crazy drowsiness, during the day I have lunch, tidy up the kitchen, if I lie down at 2 pm, if left, I'll sleep until 6 pm... Night comes, I've showered, if I lie down at 10 pm, I'll sleep until 9 am the next day, so it's a sleepiness I didn't have before, my goodness. And I don't have the will to walk, I can't stand riding the bus. Oh, and another thing... They didn't do it to me, because they say there's a way to do it, is peeing, practically, from October until... Until the beginning of this month... Every night is two diapers and a big pad. Now, it's been two weeks since it stopped. During the day, I can walk as much as I want, if I go to town I can walk without even needing a pad, just underwear, but at night... Now I'm not putting anything anymore... It's not leaking.

**2) I understand that you chose not to participate in the pulmonary rehabilitation program. Can you tell me about it?**

Response: Two very strong reasons for me: The distance is very far and I have to take two buses. From here, I take one that stops at Pinguim... Around there... On Andradas avenue, and then I have to take another. It takes time. In addition, there is no one to do things here at home. So, lunch, right!? Every day I was doing laundry because it was leaking, even with a diaper, it was leaking, I had an anti-slip plastic, but, like, I used all possible means. I cannot stand to take a bus; it bumps all the time. My bladder seems to be out of place, the doctor even said that my bladder is overactive. I said, wait a minute, there's overactive bladder too? He said, there is... Well... I didn't know any of this, but I'll ask, because I went to all the doctors, neurologist, angiologist, geriatrician, orthopedist, because... My legs hurt, but it's not the bone, I think it's the nerve. The doctor said there's a stocking now because I can't stand... It's swelling, it's still swelling... A lot of things I didn't have before being hospitalized.

**3) What was the most important factor that prevented you from participating in the pulmonary rehabilitation program?**

Response: Distance, right!? Definitely, the distance. It is a very long time, and takes almost half a day.

**4) What information did you receive about the pulmonary rehabilitation program?**

Response: No.

**5) How do you think a pulmonary rehabilitation program is?**

Response: Look... I... Personally, I thought I still had some lung sequelae too. I don't know, at home there were two things like... Breathing and lifting the legs, including raising the body from bed about 10 times with breathing.

1. **Do you think that the rehabilitation would provide any benefit for your health?**

Response: Well, it would bring because I wouldn't get so tired. Because every step of the stairs I climb still tires me out, I just don't get tired to sleep more. Because I used to, you know... That inhaler... The one we put in our mouth and inhale twice, I've been doing it until today.

1. **Do you consider participating in a pulmonary rehabilitation program in the future?**

Response: Oh, I consider, I think almost everyone needs it. Everyone who had it is complaining about something.

**8) In the future, for you to participate in a rehabilitation program, what circumstances should change?**

Response: A closer location, because I can't stand riding the bus and I can't afford an Uber, because... I receive a basic salary, right!? If I could pay, I would, to have better health I would.
